# Psychological status and physical performance are independently associated with autonomic function

**DOI:** 10.1186/s12938-022-00996-7

**Published:** 2022-05-05

**Authors:** Nur Husna Shahimi, Choon-Hian Goh, Sumaiyah Mat, Renly Lim, Vivian Ci Ai Koh, Samuel R. Nyman, Maw Pin Tan, Einly Lim

**Affiliations:** 1 Department of Biomedical Engineering, Faculty of Engineering, University of Malaya, 50603 Kuala Lumpur, Malaysia; 2 Ageing and Age-Associated Disorders Research Group, Faculty of Medicine, University of Malaya, 50603 Kuala Lumpur, Malaysia; 3 Centre for Healthy Aging and Wellness, Faculty of Health Sciences, Universiti Kebangsaan Malaysia, 50300 Kuala Lumpur, Malaysia; 4 Department of Mechatronics and BioMedical Engineering, Lee Kong Chian Faculty of Engineering and Science, Universiti Tunku Abdul Rahman, Bandar Sungai Long, 43200 Kajang, Selangor Malaysia; 5 UniSA Clinical and Health Sciences, University of South Australia, Adelaide, 5000 Australia; 6 Aevice Health Pte Ltd, Novelty BizCentre, Singapore; 7 Bournemouth University Clinical Research Unit, Department of Medical Science and Public Health, Bournemouth University, Dorset, UK

**Keywords:** Fall, Autonomic nervous system, Psychological disorder, Physical performance

## Abstract

**Background:**

Falls among older adults have become a global concern. While previous studies have established associations between autonomic function indicator; heart rate variability (HRV) and blood pressure variability (BPV) with fall recurrence, as well as physical inactivity and psychological disorders as risk factors for falls, the influence of physical activity and psychological status on autonomic dysfunction observed among older fallers has not been adequately investigated. The aim of this study was to evaluate the relationship between psychological disorder and physical performance on the autonomic nervous system (ANS) in older fallers. We hypothesised that older fallers have poorer autonomic function, greater dependency on others and were associated with psychological disorders. Furthermore, we hypothesised that both physical performance and psychological status can contribute to the worsening of the autonomic function among the elderly.

**Methods:**

In this cross-sectional survey, adults aged ≥ 60 years were recruited. Continuous non-invasive BP was monitored over 5 min of supine and 3 min of standing. Psychological status was assessed in terms of depression, anxiety, stress, and concern about falling, while functional status was measured using time-up-and-go, functional reach, handgrip and Lawton’s Instrumental Activities of Daily Life (IADL) scale.

**Results:**

A total of 62 participants were recruited consisting of 37 fallers and 25 non-fallers. Multivariate analysis revealed that Lawton IADL was independently associated with systolic blood pressure variability (SBPV) and diastolic blood pressure variability (DBPV) during both supine (SBPV: *r*^2^ = 0.080, *p* = 0.025; DBPV: *r*^2^ = 0.064, *p* = 0.046) and standing (SBPV: *r*^2^ = 0.112, *p* = 0.008; DBPV: *r*^2^ = 0.105, *p* = 0.011), while anxiety score was independently associated with SBPV and DBPV during standing (SBPV: *r*^2^ = 0.112, *p* = 0.009; DBPV: *r*^2^ = 0.105, *p* = 0.011) as compared to the other parameters.

**Conclusion:**

Our findings suggest that fallers had poorer ANS, greater dependence in IADLs, and were more anxious. IADL dependency and anxiety were the most predictive of autonomic dysfunction, and can be used in practice to identify poor autonomic function for the prevention of falls and cardiovascular diseases among older adults.

## Introduction

Falls among older adults is a major public health concern due to an increased risk of death and serious injuries [1]. Fall-related injuries may result in a loss of independence in fallers and increase their dependency on others [2]. Older adults who experience a fall-related injury are more likely to enter a long-term care facility or nursing home [3]. In Malaysia, the prevalence of falls was 14% among community-dwelling older adults aged 60 years and above in the past year [4].

The occurrence of falls in older adults is associated with disorders within the autonomic nervous system [5]. Heart rate variability (HRV) and blood pressure variability (BPV) measures have been used as indicators of autonomic function. Goh et al*.* [6] observed a reduction in beat-to-beat BPV while standing in individuals with a history of falls in the preceding year, whereas impaired autonomic function with higher sympathetic activity and reduced parasympathetic activity (as indicated by HRV) has become a predictor for recurrent falls, independent of orthostatic phenomena [7].

The occurrence of falls in older adults is associated with psychological factors. Fallers may avoid physical activity as a result of fear of falling [8, 9], with deleterious effects on cognition and mental health [8, 10]. Previous studies have reported a significant increase in the levels of anxiety and/or depression due to fear of falling among older fallers in United States [8] and Europe [11]. Bidirectional relationships between mental state and physical inactivity have also been observed among the African-American population, where depressive symptoms were more common among those with mobility limitation [12].

Physical inactivity and psychological disorders are also both associated with changes in the autonomic nervous system. Studies in older adults have reported that exercise training [13] and physical activity [14, 15] eventually enhanced the parasympathetic and overall autonomic tone regardless of age [16–18]. Furthermore, sedentarism and physical inactivity resulted in lower HRV [19], indicating impaired autonomic cardiac modulation [20]. In addition to physical inactivity, a direct correlation has been reported between autonomic function and psychiatric illness, such as major depressive disorder, panic disorder, and anxiety [21–24]. Impairment in autonomic function, as reflected by both HRV and BPV measures, has also been linked to an increase in cardiovascular disease among psychiatric patients [25–27].

While previous studies have established associations between HRV and BPV with fall recurrence, as well as physical inactivity and psychological disorders as risk factors for falls, the influence of physical activity and psychological status on autonomic dysfunction observed among older fallers has not been adequately investigated. Furthermore, we are not aware of any study which investigated the association between physical performance and autonomic dysfunction. Identification of measures of physical activity, physical performance and psychological status which best predict changes in autonomic function among older fallers would help identify patients with increased risk of recurrent falls and cardiovascular diseases. Therefore, our study aims are twofold: (i) to compare the psychological function, physical activity and physical performance between fallers and non-fallers among older adults, and (ii) to determine the associations between psychological function, physical activity and physical performance with the autonomic nervous system.

## Results

A total of 62 participants were recruited in the study. Of those, 37 (59.68%) had at least one fall in the past 2 months and 25 (40.32%) had no history of falls in the past year (Table 1).

**Table 1:** Characteristics of participants

	Fall in the past 2 months		*p*-value
	Yes (*N* = 37)	No (*N* = 25)	
Age, years (mean ± s.d.)	74.26 ± 8.86	70.88 ± 7.22	0.116
Body mass index, kg/m2, (mean ± s.d.)	24.85 ± 5	23.43 ± 5.58	0.297
Gender, female, *n* (%)	22 (57.9)	17 (68.0)	0.427
Ethnicity, *n* (%)			0.859
Malay	8 (21.1)	5 (20.0)	
Chinese	24 (63.2)	17 (68.0)	
Indian	6 (15.8)	3 (12.0)	
Education, *n* (%)			0.161
No formal	3 (8.1)	2 (8.3)	
Primary	9 (24.3)	2 (8.3)	
Secondary	15 (40.5)	9 (37.5)	
Tertiary	10 (27.0)	11 (45.8)	
Physical performance (mean ± s.d.)			
Time-up-and-go (TUG) test	23.23 ± 18.45	14.66 ± 13.06	0.049^*^
Functional reach	23 ± 7.76	26.04 ± 7.55	0.130
Hand grip strength (right)	16.28 ± 5.2	20.91 ± 7.03	0.004*
Hand grip strength (left)	14.22 ± 5.51	19.57 ± 6.75	0.001**
Psychological status (mean ± s.d.)			
DASS-21 Depression	4.65 ± 6.73	1.84 ± 3.95	0.043*
DASS-21 Stress	6.32 ± 7.73	3.84 ± 5.59	0.173
DASS-21 Anxiety	5.24 ± 5.38	2.56 ± 4.38	0.043*
FES-1	13. 34 ± 5.7	9.84 ± 3.65	0.004*
Physical activity (mean ± s.d.)			
PASE	64.05 ± 52.7	114.20 ± 48.25	< 0.001**
LAWTON	5.32 ± 2.86	7.32 ± 1.57	0.001**
Baseline haemodynamic parameters (mmHg)			
Supine SBP	120.48 ± 19.1	107.9 ± 15.49	0.008*
Supine DBP	77.2 ± 16.1	72.92 ± 11.71	0.257
Supine HR	71.58 ± 11	68.92 ± 9.48	0.326

*DASS-21* Depression Anxiety Stress Scale 21, *FES-1* Falls Efficacy Scale-1, *PASE* Physical Activity Scale for the Elderly, *LAWTON* Lawton Instrumental Activities of Daily Living Scale, *SBP* systolic blood pressure, *DBP* diastolic blood pressure, *HR* heart rate

^*^Independent *T*-test *p*-value < 0.05

^**^Independent *T*-test *p*-value < 0.001

### Psychological function and physical performance in fallers versus non-fallers

There were significant differences between fallers and non-fallers, with fallers having worse scores in physical performance (TUG test, left and right-hand grip strength, PASE and LAWTON), depression and anxiety (DASS-21), concern about falling (FES-1), and supine rest systolic blood pressure (SBP).

### Autonomic function indices in fallers versus non-fallers

**Fig. 1:**
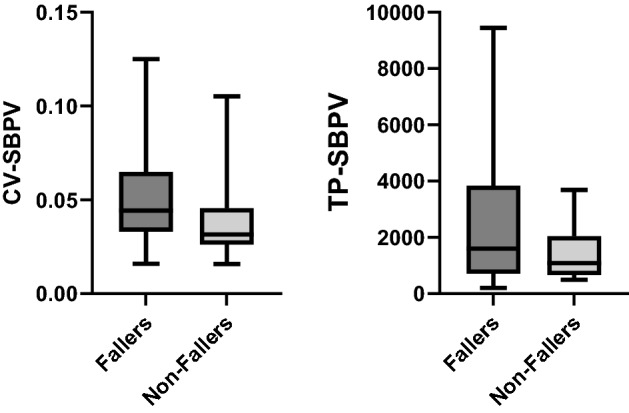
Significant findings of BPV indices at supine rest in fallers and non-fallers

**Fig. 2:**
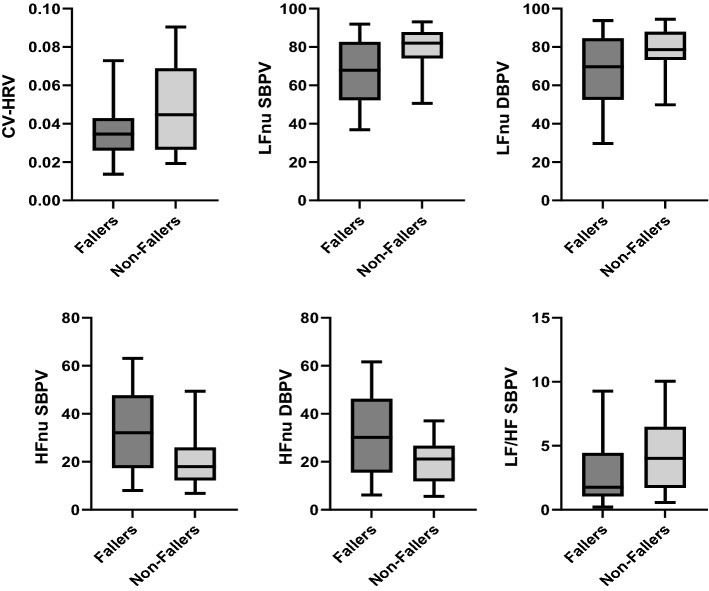
Significant findings of HRV and BPV indices at standing in fallers and non-fallers

Tables 2 and 3 summarise heart rate variability (HRV) and blood pressure variability (BPV) indices in fallers and non-fallers, respectively, at the supine and upright positions. During supine rest, SBPV-CV and SBPV-TP were significantly higher in fallers than non-fallers. This may indicate poorer autonomic function in fallers as compared to non-fallers. While standing, HF-nu of both SBPV and DBPV were significantly higher in fallers compared to non-fallers. The converse was observed for HRV-CV and LF-nu for both SBPV and DBPV as well as SBPV-LF/HF, which were lower in fallers than non-fallers. The findings that were significant have been displayed in the form of box charts (refer Figs. 1 and 2).

**Table 2:** HRV and BPV indices at supine rest in fallers and non-fallers

	Heart rate			Systolic blood pressure			Diastolic blood pressure		
	Fallers (*N* = 37)	Non-fallers (*N* = 25)	*p*-value	Fallers (*N* = 37)	Non-fallers (*N* = 25)	*p*-value	Fallers (*N* = 37)	Non-fallers (*N* = 25)	*p*-value
Time domain indices (mean ± s.d.)									
SDNN	29.28 ± 16.01	35.47± 19.44	0.173	4.71 ± 3.22	3.41 ± 1.51	0.065	3.06 ± 2.06	2.56 ± 1.22	0.282
CV	0.033 ± 0.015	0.038 ± 0.019	0.162	0.051 ± 0.04	0.038 ± 0.02	0.05*	0.057 ± 0.036	0.043 ± 0.023	0.076
ARV	18.20 ± 13.05	23.32 ± 19.88	0.221	1.125 ± 0.64	0.89 ± 0.25	0.063	0.88 ± 0.42	0.73 ± 0.344	0.122
RMSRV	24.16 ± 16.99	31.46 ± 27.29	0.195	1.75 ± 0.96	1.38 ± 0.42	0.071	1.43 ± 0.70	1.18 ± 0.52	0.120
Frequency domain indices (mean ± s.d.)									
LF-nu	51.91 ± 19.41	47.87 ± 17.84	0.411	65.56 ± 18.83	68.06 ± 14.07	0.574	66.41 ± 16.92	69.44 ± 13.96	0.461
HF-nu	48.1 ± 19.41	52.12 ± 17.84	0.411	34.44 ± 18.83	31.94 ± 14.07	0.574	33.59 ± 16.91	30.55 ± 13.96	0.461
TP	190,771 ± 113,574	293,909 ± 137,744	0.089	2700 ± 4777.8	1556.1 ± 1115.8	0.044*	1845.1 ± 2842.2	971.6 ± 699.3	0.138
LF/HF	1.504 ± 1.26	1.18 ± 0.86	0.265	2.88 ± 2.21	2.68 ± 1.43	0.696	3.08 ± 2.69	3.03 ± 1.93	0.931

*SDNN* standard deviation of NN interval, *CV* coefficient variation, *ARV* average real variability, *RMSRV* root mean square of real variability, *LF-nu* low-frequency normalised unit, *HF-nu* high-frequency normalised unit, *TP* total power, *LF/HF* ratio of LF and HF

^*^Independent *T*-test *p*-value < 0.05

**Table 3:** HRV and BPV indices at the standing position in fallers and non-fallers

	Heart rate			Systolic blood pressure			Diastolic blood pressure		
	Fallers (*N* = 35)	Non-fallers (*N* = 25)	*p*-value	Fallers (*N* = 35)	Non-fallers (*N *= 25)	*p*-value	Fallers (*N* = 35)	Non-fallers (*N* = 25)	*p*-value
Time domain indices (mean ± s.d.)									
SDNN	30.93 ± 17.6	40.23 ± 21.89	0.072	5.54 ± 2.44	5.47 ± 2.19	0.921	3.88 ± 1.89	3.75 ± 1.64	0.772
CV	0.036 ± 0.018	0.048 ± 0.023	0.008**	0.068 ± 0.041	0.055 ± 0.024	0.168	0.059 ± 0.04	0.05 ± 0.021	0.1977
ARV	15.27 ± 10.64	19.91 ± 19.15	0.230	1.15 ± 0.49	1.10 ± 0.63	0.745	0.87 ± 0.34	0.88 ± 0.43	0.897
RMSRV	22.79 ± 20.73	27.99 ± 27.95	0.407	1.97 ± 1.09	1.74 ± 1.11	0.417	1.42 ± 0.60	1.40 ± 0.67	0.905
Frequency domain indices (mean ± s.d.)									
LF-nu	52.69 ± 18.67	53.5 ± 22.89)	0.882	66.95 ± 18.68	78.33 ± 15.37	0.008**	67.02 ± 17.7	78.25 ± 14.5	0.008**
HF-nu	47.30 ± 18.67	46.50 ± 22.89	0.882	33.05 ± 18.68	21.67 ± 15.37	0.008**	31.88 ± 17.7	20.52 ± 14.4	0.005**
TP	86,070 ± 113,574	414,905 ± 505,243	0.288	1421.7 ± 1190.1	1536.6 ± 2322	0.803	785.8 ± 836.2	732.3 ± 664.7	0.792
LF/HF	1.511 ± 1.137	1.898 ± 1.807	0.313	2.92 ± 2.81	4.42 ± 3.56	0.033*	3.63 ± 3.68	4.95 ± 3.83	0.184

*SDNN* standard deviation of NN interval, *CV* coefficient variation, *ARV* average real variability, *RMSRV* root mean square of real variability, *LF-nu* low-frequency normalised unit, *HF-nu* high-frequency normalised unit, *TP* total power, *LF/HF* ratio of LF and HF

^*^Independent *T*-test *p*-value < 0.05

^**^Independent *T*-test *p*-value < 0.001

### Bivariate Pearson’s correlation analyses

Table 4 summarises the relationship between psychological variables and both time domain and spectral analysis indices of autonomic function for fallers, non-fallers and all participants during supine rest. Overall, among the stress, depression and anxiety scores extracted from the DASS-21 questionnaire, only anxiety and stress score were correlated with autonomic function indices during supine rest. Significant correlation was observed between anxiety and DBPV-CV in non-fallers as well as stress score and the CV index for both SBPV and DBPV.

**Table 4:** Bivariate correlation between psychological disorder and autonomic function indices according to supine in both group of fallers and non-fallers (combined), fallers and non-fallers

	Supine rest (*n* = 62)								
	DASS-21 Depression			DASS-21 Anxiety			DASS-21 Stress		
	Combined	Fallers	Non-fallers	Combined	Fallers	Non-fallers	Combined	Fallers	Non-fallers
Time domain									
Heart rate variability									
SDNN	− 0.086	0.008	− 0.179	− 0.066	0.068	− 0.165	0.060	0.100	0.085
CV	− 0.030	0.078	− 0.124	− 0.070	0.094	− 0.206	0.088	0.160	0.071
ARV	− 0.134	− 0.054	− 0.212	− 0.107	− 0.026	− 0.132	0.004	0.021	0.052
RMSRV	− 0.079	0.010	− 0.140	− 0.071	0.043	− 0.118	0.038	0.066	0.086
Systolic blood pressure variability									
SDNN	0.041	− 0.017	0.017	− 0.110	− 0.149	− 0.317	0.242	0.289	− 0.117
CV	0.062	− 0.002	0.042	0.028	0.057	− 0.393	0.388**	0.502**	− 0.214
ARV	− 0.045	− 0.130	0.014	− 0.164	− 0.296	− 0.049	− 0.014	− 0.094	0.106
RMSRV	− 0.041	− 0.143	0.171	− 0.140	− 0.283	0.079	− 0.005	− 0.089	0.163
Diastolic blood pressure variability									
SDNN	0.072	0.015	0.171	− 0.102	− 0.110	− 0.233	0.246	0.306	− 0.029
CV	0.070	− 0.012	0.151	0.011	0.080	− 0.397*	0.339**	0.482**	− 0.212
ARV	− 0.026	− 0.118	0.066	− 0.122	− 0.242	− 0.035	− 0.029	− 0.137	0.123
RMSRV	− 0.002	− 0.157	0.327	− 0.065	− 0.234	0.168	− 0.004	− 0.148	0.270
Spectral analysis									
Heart rate variability									
LF-nu	0.123	0.103	0.099	0.193	0.279	− 0.008	0.090	0.152	− 0.096
HF-nu	− 0.123	− 0.103	− 0.099	− 0.193	− 0.279	0.008	− 0.090	− 0.152	0.096
TP	− 0.119	− 0.046	− 0.118	− 0.090	0.089	− 0.130	0.050	0.099	0.112
LF/HF	0.149	0.123	0.098	0.170	0.234	− 0.072	0.103	0.145	− 0.108
Systolic blood pressure variability									
LF-nu	0.078	0.058	0.240	0.119	0.199	− 0.264	− 0.001	0.058	− 0.114
HF-nu	− 0.078	− 0.058	− 0.240	− 0.119	− 0.199	0.022	0.001	− 0.058	0.114
TP	− 0.041	− 0.130	0.070	0.080	0.047	− 0.022	− 0.011	− 0.048	− 0.155
LF/HF	0.020	− 0.044	0.210	0.193	0.298	− 0.161	− 0.005	0.028	− 0.142
Diastolic blood pressure variability									
LF-nu	0.073	0.009	0.104	0.131	0.178	0.127	0.053	0.113	− 0.037
HF-nu	− 0.073	− 0.099	− 0.104	− 0.131	− 0.178	− 0.127	− 0.053	− 0.113	0.037
TP	− 0.027	− 0.114	0.352	0.096	0.059	0.096	− 0.028	− 0.086	0.147
LF/HF	− 0.009	− 0.014	− 0.009	0.146	0.187	0.065	0.092	0.157	− 0.095

*SDNN* standard deviation of NN interval, *CV* coefficient variation, *ARV* average real variability, *RMSRV* root mean square of real variability, *LF-nu* low-frequency normalised unit, *HF-nu* high-frequency normalised unit, *TP* total power, *LF/HF* ratio of LF and HF

^*^Pearson’s correlation significant at *p*-value < 0.05

^**^Pearson’s correlation significant at *p*-value < 0.001

**Fig. 3:**
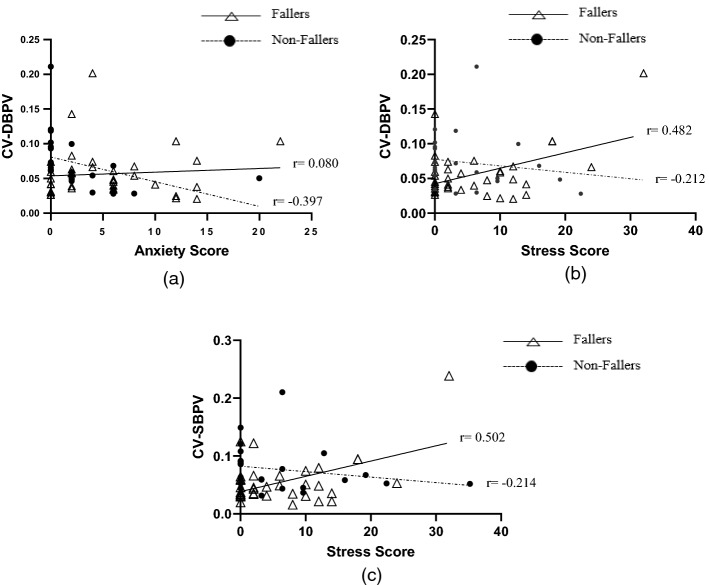
Relationship between **a** CV-DBPV and DASS-21 anxiety score, **b** CV-DBPV and DASS-21 stress score, and **c** CV-SBPV and DASS-21 stress score during supine in both group of fallers and non-fallers

**Fig. 4:**
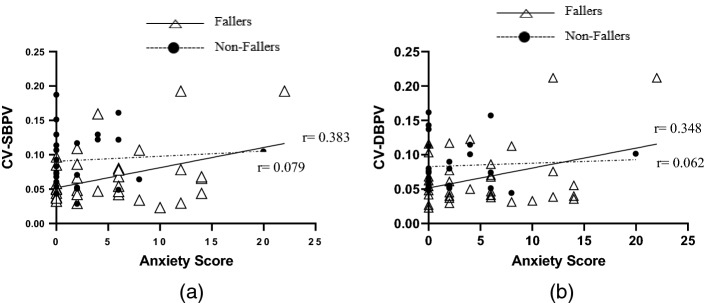
Relationship between **a** CV-SBPV and DASS-21 anxiety score, and **b** CV-DBPV and DASS-21 anxiety score during standing in both group of fallers and non-fallers

At the standing position (refer Table 5), significant correlation existed between stress scores and SBPV-RMSRV as well as DBPV-CV. Moreover, there was a significant correlation between depression and HRV-SDNN. Conversely, anxiety scores correlated significantly with time domain (i.e. SBPV-CV and DBPV-CV) indices of autonomic function only. The significant findings from the bivariate correlations are displayed in the form of scatter plots (refer Figs. 3 and 4).

**Table 5:** Bivariate correlation between psychological disorder and autonomic function indices according to standing in both group of fallers and non-fallers (combined), fallers and non-fallers

	Standing position (*n* = 60)								
	DASS-21 Depression			DASS-21 Anxiety			DASS-21 Stress		
	Combined	Fallers	Non-fallers	Combined	Fallers	Non-fallers	Combined	Fallers	Non-fallers
Time domain									
Heart rate variability									
SDNN	− 0.258*	− 0.175	− 0.332	− 0.249	− 0.124	− 0.312	0.020	0.147	− 0.056
CV	− 0.232	− 0.122	− 0.325	− 0.250	− 0.058	− 0.284	0.015	0.192	− 0.111
ARV	− 0.213	− 0.190	− 0.239	− 0.154	− 0.080	− 0.166	0.011	0.060	0.022
RMSRV	− 0.176	− 0.146	− 0.198	− 0.193	− 0.181	− 0.160	0.086	0.172	0.037
Systolic blood pressure variability									
SDNN	− 0.038	− 0.074	0.035	0.085	0.004	0.239	− 0.007	− 0.069	0.097
CV	0.109	0.037	0.214	0.335**	0.383*	0.079	0.211	0.253	0.019
ARV	− 0.059	− 0.012	− 0.193	0.033	− 0.080	0.171	0.141	− 0.041	0.373
RMSRV	− 0.020	0.025	− 0.216	0.251	0.289	0.145	0.281*	0.247	0.311
Diastolic blood pressure variability									
SDNN	0.066	0.017	0.177	0.083	− 0.029	0.287	0.100	0.061	0.158
CV	0.141	0.054	0.300	0.325*	0.348*	0.062	0.268*	0.315	0.021
ARV	− 0.071	− 0.019	− 0.174	− 0.040	− 0.184	0.175	0.064	− 0.082	0.273
RMSRV	− 0.075	− 0.038	− 0.181	0.028	− 0.051	0.152	0.097	− 0.006	0.252
Spectral analysis									
Heart rate variability									
LF-nu	0.047	0.095	− 0.021	0.145	0.163	0.147	− 0.064	− 0.060	− 0.065
HF-nu	− 0.047	− 0.095	0.021	− 0.145	− 0.163	− 0.147	0.064	0.060	0.065
TP	− 0.201	− 0.170	− 0.206	− 0.208	− 0.180	− 0.173	0.078	0.155	0.037
LF/HF	0.032	0.103	0.025	0.069	0.087	0.129	− 0.059	− 0.071	− 0.020
Systolic blood pressure variability									
LF-nu	− 0.150	− 0.181	0.187	− 0.118	− 0.072	0.001	− 0.249	− 0.133	− 0.390
HF-nu	0.150	0.181	− 0.187	0.118	0.072	− 0.001	0.249	0.133	0.390
TP	− 0.011	0.034	− 0.055	0.028	− 0.009	0.076	0.022	0.001	0.050
LF/HF	− 0.077	− 0.056	0.079	− 0.077	− 0.051	0.053	− 0.151	− 0.012	− 0.252
Diastolic blood pressure variability									
LF-nu	− 0.132	− 0.150	0.151	− 0.103	− 0.066	0.014	− 0.182	− 0.103	− 0.251
HF-nu	0.132	0.150	− 0.151	0.103	0.066	− 0.014	0.182	0.103	0.251
TP	0.056	0.043	0.071	0.009	− 0.177	0.333	0.084	0.025	0.190
LF/HF	− 0.027	0.027	− 0.021	0.043	0.090	0.088	− 0.140	− 0.044	− 0.236

*SDNN* standard deviation of NN interval, *CV* coefficient variation, *ARV* average real variability, *RMSRV* root mean square of real variability, *LF-nu* low-frequency normalised unit, *HF-nu* high-frequency normalised unit, *TP* total power, *LF/HF* ratio of LF and HF

^*^Pearson’s correlation significant at *p-*value < 0.05

^**^Pearson’s correlation significant at *p*-value < 0.001

### Factors independently associated with autonomic function

Factors associated with autonomic nervous system (ANS) function were tested using variables that showed significant differences between the fallers and non-fallers groups (Table 1). These include history of falls, psychological disorder (DASS-21 Depression, DASS-21 Anxiety, FES-1) and physical performance (TUG, Lawton IADL). The CV index for both SBPV and DBPV were selected to represent ANS as it shows consistent significant correlation in SBPV and DBPV while standing and at supine rest.

Overall, Table 6 contains models for both systolic and diastolic blood pressure variability during supine rest and standing. History of falls (*R*^2^ = 6.5%) and Lawton IADL scores (*R*^2^ = 8.0%) were found to be independently associated with systolic BPV during supine rest, but not DASS-21 depression and DASS-21 anxiety scores, FES-1, as well as TUG. Standing systolic blood pressure variability (SBPV)-CV was independently associated with DASS-21 anxiety score (*R*^2^ = 11.2%) and Lawton IADL score (*R*^2^ = 11.2%), but not history of falls, DASS-21 depression, FES-1 and TUG. Supine diastolic blood pressure variability (DBPV)-CV was independently associated with Lawton IADL score (*R*^2^ = 6.4%), while the remaining variables were no longer significant. Standing diastolic blood pressure variability (DBPV)-CV was independently associated with DASS-21 anxiety (*R*^2^ = 10.5%) and Lawton IADL scores (*R*^2^ = 10.5%), but not history of falls, DASS-21 depression, FES-1 and TUG. According to the percentage of R-squared between two variables, Lawton IADL has a better correlation in predicting autonomic dysfunction during supine rest and standing, while anxiety score has a better correlation with autonomic dysfunction during standing. To summarise, a higher *R*^2^ value indicates a stronger predictive value between two variables. Generally, the adjusted *R*^2^ values were relatively small although significant associations were observed between DASS-21 anxiety, Lawton IADL and BPV.

**Table 6:** Factors independently associated with autonomic function

Variables	Mean difference (95% confidence interval)							
	Supine rest				Standing position			
	CV-SBP	*R*^2^	CV-DBP	*R*^2^	CV-SBP	*R*^2^	CV-DBP	*R*^2^
Falls history	**0.018 (0.000 to 0.035)**	0.065	0.015 (− 0.002 to 0.031)	0.051	0.013 (− 0.006 to 0.031)	0.032	0.017 (− 0.002 to 0.037)	0.051
Depression score	0.000 (− 0.001 to 0.002)	0.004	0.000 (− 0.001 to 0.002)	0.005	0.001 (− 0.001 to 0.002)	0.012	0.001 (− 0.001 to 0.003)	0.020
Anxiety score	0.000 (− 0.002 to 0.002)	0.001	0.000 (− 0.002 to 0.002)	0.000	**0.002 (0.001 to 0.004)**	0.112	**0.002 (0.001 to 0.004)**	0.105
FES− 1	0.001 (− 0.001 to 0.003)	0.026	0.001 (− 0.001 to 0.002)	0.011	0.001 (− 0.001 to 0.003)	0.017	0.001 (0.000 to 0.003)	0.040
TUG	0.000 (0.000 to 0.001)	0.046	0.000 (0.000 to 0.001)	0.035	0.000 (0.000 to 0.001)	0.014	0.000 (0.000 to 0.001)	0.013
Lawton IADL	**− 0.004 (− 0.007 to 0.000)**	0.080	**− 0.003 (− 0.006 to 0.000)**	0.064	**− 0.005 (− 0.008 to − 0.001)**	0.112	**− 0.005 (− 0.008 to − 0.001)**	0.105
Adjusted *R*^2^	0.014		− 0.031		0.110		0.081	

*SBP* systolic blood pressure, *DBP* diastolic blood pressure, *CV* coefficient variation, *CI* confidence interval, *R*^*2*^ R-squared, *FES-1* Falls Efficacy Scale-1, *TUG* Time-up-and-go, *Lawton IADL* Lawton Instrumental Activities of Daily Living Scale

Bold values indicate *p*-value < 0.05

## Discussion

Our study found that community-dwelling older adults who experienced a fall in the past 2 months had poorer ANS function, anxiety, and were more dependent in instrumental activities of daily living.

Previous studies have also reported fear-related activity avoidance and increased feelings of anxiety and symptoms of depression among older fallers [8, 10, 28]. Fallers may develop fear of falling that further impede their physical activity and reduce mobility [29–31], muscular strength, flexibility, gait and fitness, ultimately worsening their physical performance and increasing future fall risk [32, 33]. Furthermore, fall-related injuries resulted in the inability to socialise and mobilise, leading to increased dependency on family caregivers to maintain mobility, with additional deleterious effects on psychological well-being among fallers [34].

In the supine position, significantly higher time and frequency domain were observed among fallers. These observations reflected an impairment in sympathetic and parasympathetic branches (sympathovagal balance) as well as blunted baroreflex sensitivity [35–37] among fallers. On the contrary, fallers demonstrated lower low frequency to high frequency (LF/HF) and low-frequency (LF) SBPV during upright posture, which may suggest a reduction in BP control reactivity during standing [6]. This may be associated with age-related conditions such as arterial stiffness or autonomic dysfunction [38]. Similar findings have also been observed in a previous study [6]. A reduction in BP control during standing could explain the susceptibility to falls among the fallers group, as fall events are usually associated with reduced ability to maintain the centre of gravity.

To date, we are not aware of any other study which has investigated the relationship between the psychological system and ANS function among fallers at different postures. Our multivariate analysis results showed that anxiety is an independent predictor for ANS function in the standing position. Several studies have highlighted that higher anxiety scores were associated with higher sympathetic reaction, as represented by both beat-to-beat BPV [39, 40] and long-term BPV [41]. A study by Piccirillo et al*.* [39] showed that tilt induced a significant increase of systolic blood pressure in participants with severe anxiety (i.e. having two or more anxiety symptoms), but no significant change was observed in participants without any anxiety symptoms. On the other hand, participants with moderate (i.e. having single anxiety symptom) or severe anxiety demonstrated significantly higher diastolic blood pressure variability during tilt, as compared to healthy participants [39]. This may be explained by the augmentation of the anxiety state (or psychological hyperarousal) in patients with higher anxiety level during tilt, which led to an increase in their sympathetic reaction [40].

Numerous studies have been performed to investigate the bidirectional relationship between physical inactivity and cardiac autonomic modulation [42–44]. However, no comparison among the different physical activity measures has been made to identify which best reflects autonomic response. Our results showed that Lawton IADL, a self-reported questionnaire which detects functional dependence in instrumental activities of daily living, was the only independently associated factor for autonomic function. Lawton IADL is closely associated with decline in physical function, which in turn increases sedentary behaviour. Sedentary behaviour would then lead to reduced stimulation of the baroreflex mechanisms, leading to sympathetic predominance and a reduction in vagal activity at rest [42]. In contrast, according to Tornberg et al*.* [45], an active lifestyle among adults is associated with consistent increase in cardiorespiratory physical fitness and lower heart rate while increasing left ventricle filling, venous return, and stroke volume, which is strongly associated to increased parasympathetic activity of the heart at rest.

Nonetheless, our results showed that physical performance tests, such as time-up-and-go, functional reach and handgrip strength, were not independent predictors of autonomic function indices. Physical performance tests only provide snapshots measures of the performance of specific body parts, such as muscle strength, walking speed and dynamic balance, and therefore are not reliable indicators of overall functional ability.

### Clinical implications

The ageing population is growing, and the potential problems associated with ageing include cardiovascular diseases and injuries caused by falls. As these complications are closely related to autonomic function, clinicians have frequently adopted the tilt-table test (i.e. an autonomic function test) to detect any abnormality in the autonomic function by observing blood pressure and heart rate changes in the patients during the test. However, this requires the use of non-invasive, beat-to-beat haemodynamic monitoring technologies, which are not widely available in all clinical centres. In addition, it requires the patients to travel to the designated clinical centres, which may cause weariness and stress among older adults and their caregivers. In the tilt table/sit-to-stand assessment, the patients are required to stand upright after lying down for several minutes, which may be challenging for frail older adults.

A pre-screening assessment which could help identify patients with an increased risk of autonomic dysfunction is therefore useful for early intervention. Our findings suggest that validated questionnaires such as DASS-21 and LAWTON could serve as pre-screening tools to identify patients with autonomic dysfunction. As these questionnaires are freely available online, virtual communication between physicians and patients can be conducted, thus reducing the time taken for travel to clinical centres for autonomic function tests. This could aid in the diagnosis of autonomic dysfunction severity and reduce the risk of future falls and cardiovascular diseases.

## Limitations and future considerations

As available data only studied the cross-sectional relationship between autonomic function and psychological and functional status, we are unable to establish causation or define any temporal relationship. In addition, many factors which may influence the variability in postural changes in blood pressure and heart rate remain unaccounted for as potential factors such as medication use had not been considered in this study. While DASS-21 and LAWTON were shown to be predictive of autonomic dysfunction, the small adjusted R^2^ values suggested that the haemodynamic parameters evaluated only accounted for a small amount of the variability in the above outcomes. However, using the rule of thumb of multivariate regression, with an absolute minimum of 10 participants per predictor variables, the current study had sufficient sample for the number of parameters input into the multivariate regression. The autonomic parameters have the potential to detect psychological problems in the older population, which currently remains underdiagnosed. Future studies should involve larger sample sizes to ascertain the usefulness of these parameters. Furthermore, the processing length for the standing signals is only 3 min, which is known to be shorter for the analysis of cardiac diseases. However, the limitation of the length is due to the active standing protocol where the current study needs to observe the heart rate and blood pressure changes within 3-min standing. Although a significant correlation was observed between mental state and shorter ECG recordings (< 1 min) in previously published studies, further work should take into account the length of the signals carried out to determine the potential role of ANS function as a biomarker of psychological issues related to falls as well as interventions which enhance ANS to modify falls risk, or ANS as biofeedback for fall prevention interventions.

## Conclusion

Our findings revealed that CV-SBPV at supine rest was independently associated with falls in the preceding two months, with differences in CV-DBPV at the standing position accounted for by functional ability. Particularly in fallers, significant correlations existed between stress and anxiety with BPV. By knowing the association between these modifiable risk factors and autonomic function, early identification could be done of patients at risk of recurrent falls and cardiovascular diseases and thus timely intervention can be performed.

## Methods

### Design and study population

This was an exploratory cross-sectional study involving 37 fallers and 25 non-fallers, recruited between October 2019 and October 2020. Falls were defined as “unintentionally coming to rest on the ground, floor or other lower level” [46]. Adults aged 60 years and above who had a fall injury were recruited from an emergency department, primary care clinics and geriatric outpatients as well as referrals from other departments and specialists at University Malaya Medical Center. Only mobile individuals with at least one fall in the past two months were included. Age-matched healthy volunteers with no history of falls (non-fallers) were recruited from the patient’s family members, spouse or word of mouth advertising. This study protocol was approved by a University Research Ethics Committee (MREC ID No: 2019525-7445) prior to commencement. Written informed consent was obtained from all participants.

### Data collection

Characteristics including age, gender, characteristics of falls, past medical history, medication review, anthropometric measurements (height, weight, waist and hip ratio) and continuous blood pressure (BP) and heart rate (HR) assessments were obtained from all participants.

### Outcome measures

The outcome measures used in this study include measures on physical performance, physical activity and psychological function.

#### Physical performance

We conducted three types of tests to assess the individual’s physical performance: timed-up-and-go test, functional reach and handgrip strength. Timed-up-and-go test evaluated the time taken to rise from the sitting position on a standard armchair, walk for 3 m, turn back, and sit down again. Longer durations taken by the participants to complete the task indicate poorer physical performance [47]. Functional reach was measured by asking the participants to stand with feet shoulder-width apart with the right hand outstretched in a maximal forward reach, while maintaining a fixed base of support [48]. The distance between the positions of the third distal interphalangeal joint when the participants were at the upright position and then at maximal forward stretch was calculated in centimetres (cm). A longer stretch distance indicates a better balance outcome. Both tests have been widely used to assess physical function, risk of falling and frailty among older adults [49]. Lastly, muscle strength was assessed with the handgrip strength test, conducted using the Jamar digital hand grip dynamometer (Sammons Preston, Bolingbrook, IL, USA). The average of three measurements obtained for both right and left hand with the elbow flexed at 90°, was calculated in centimetres [50].

#### Physical activity

Instrumental activities of daily living were assessed using the Lawton’s Instrumental Activities of Daily Life (IADL) scale [51]. This scale measures functional independence by recording the ability of the participants to perform the activities: use of telephone, shopping, use of transportation, food preparation, doing housework, managing own laundry, managing medication and ability to handle finances. The maximal score of this scale is eight and a lower score indicates increased functional dependence. Next, Physical Activity Scale for the Elderly (PASE) was used to assess current level of physical activity [29]. Test items reflected leisure, domestic life and work. Participants were required to answer the questions on frequency (“none”, “seldom”, “sometimes” and “often”) as well as duration (“less than 1 h”, “1–2 h”, “2–4 h” and “more than 4 h”) of each activity. Higher scores indicate higher activity levels.

#### Psychological status

Depression, anxiety and stress were measured with the 21-item Depression, Anxiety, Stress Scale (DASS-21). Participants were required to self-report the frequency and severity of the negative emotions of depression, anxiety and stress over the previous week. The frequency and severity ratings are based on a 4-point Likert scale, with 0 indicating “did not apply to me at all” and 3 indicating “applied to me very much, or most of the time”. The scores were calculated individually for the three components of depression, anxiety and stress [52]. In addition, the Falls Efficacy Scale International (FES-1) short form was assessed. This recorded concern with falling while getting dressed or undressed, taking bath or shower, getting in or out of a chair, going up or down stairs, reaching for something above the head or the ground, walking up or down a slope and going out for social event scored on a 4-point Likert scale. Higher scores indicate higher concern for falling, with a maximal score of 28 [53].

## Beat-to-beat heart rate and blood pressure assessment

Each participant was required to undergo a supine-to-standing orthostatic test with non-invasive beat-to-beat heart rate (HR) and blood pressure (BP) monitoring obtained using the vascular unloading technique (Task Force, CNSystem, Austria). R–R intervals were derived based on the electrocardiogram (ECG) signal (sampling rate = 1 kHz), while beat-to-beat blood pressure values were derived from the fingertip photoplethysmographic (PPG) signal (sampling rate = 100 Hz), with the signal processing details described in the following section. Participants were required to lie in the supine position for at least 10 min before the 3-min active stand test was started, with the appropriately sized finger cuff attached on the middle and index fingers [6]. All participants were refrained from talking and moving during the assessment to reduce artefacts and noise in the signals. All recordings were performed during spontaneous breathing. The presence of any symptoms of dizziness during standing was recorded.

### Signal processing

The continuous HR and BP signals for each participant were exported to MATLAB software to be processed (Version R2014b, MathWorks Inc., Natick, Massachusetts, United States). The ECG and finger blood pressure waveforms were pre-processed using custom written algorithms, where filtering, tracing, and denoising were performed to remove any unwanted artefacts. The QRS peaks of the ECG as well as the peaks/troughs of the cyclical blood pressure waveform within every cardiac cycle were then detected using standard derivative or threshold algorithms for the purpose of estimating heart rate and systolic or diastolic blood pressure. Beat-to-beat HR and BP readings were identified and separated into three different segments: supine rest, interval and standing. Blood pressure variability (BPV) and heart rate variability (HRV) were computed as time domain indices and power spectral indices.

Time domain analysis measures dispersion of blood pressure values over a given time window, while frequency domain analysis measures blood pressure fluctuation as a function of frequency. Time domain indices are divided into two categories: (1) measures of dispersion of average values over a given time window of 10-min supine rest and 3-min standing, which include standard deviation (SD) and coefficient of variation (CV); (2) estimation that accounts for the sequence of measurements over time, which include average real variability (ARV) and root mean square of real variability (RMSRV). On the other hand, frequency domain indices are obtained through spectral analysis techniques, in which we measured fluctuations in beat-to-beat blood pressure as a function of frequency. Linear detrending technique was first performed, followed by the application of fast Fourier transform (FFT) algorithms on the extracted beat-to-beat heart rate and systolic/diastolic blood pressure data to obtain the frequency domain HRV and BPV indices: low-frequency normalised unit (LFnu, HRV: 0.04–0.15 Hz, BPV: 0.07–0.14 Hz), high-frequency normalised unit (HFnu, HRV: 0.15–0.4 Hz, BPV: 0.14–0.35 Hz), total power (TP), and ratio of low-frequency and high-frequency (LF/HF) [6, 7]. The high-frequency component measures the parasympathetic activity, while the low-frequency component measures the sympathetic activity [6, 7]. The ratio of the low- to high-frequency components reflects the sympathovagal balance between the sympathetic and the parasympathetic activities [6].

### Data analysis

A priori sample size calculations were performed using G*Power statistical software [54], and with an effect size of 0.75 at a power of 80%, a minimum of 62 participants were required [55]. In addition, using the general rule of thumb described by Wilson Van Voorhis and Morgan [56], no fewer than 50 participants are required for a correlation or regression, and an absolute minimum of 10 participants per predictor variable is required for regression equations with six or more predictor variables. Therefore, with six predictors used in this study, we needed a sample size of at least 60 people. As a result, during data collection, 37 fallers and 25 non-fallers were included. Statistical analysis was conducted using the SPSS V23 statistical software (SPSS Inc, Chicago, IL, USA). Descriptive statistics were presented as mean ± standard deviation for normally distributed continuous variables and frequencies with percentages for categorical data. The difference between the groups of fallers and non-fallers in their demographic characteristics, autonomic response and psychological disorder was determined using the independent *t-*test for normally distributed continuous variables. For non-normally distributed data, continuous variables were expressed as median with quartile 1 to quartile 3 in parenthesis and the differences between groups were analysed with the Mann–Whitney *U* test. Then, correlation between psychological disorder and autonomic response was measured using the Pearson’s correlation coefficient. Finally, multivariable linear regression was used to adjust for potential confounding variables and to determine potential predictors for autonomic dysfunction. The assumptions of linear regression were checked, and all met for normality, linearity and homoscedasticity. A *p*-value < 0.05 was considered statistically significant.

## Data Availability

All relevant data are contained within the article.

## Abbreviations

ANS: Autonomic nervous system
BPV: Blood pressure variability
SBPV: Systolic blood pressure variability
DBPV: Diastolic blood pressure variability
HRV: Heart rate variability
DASS-21: Depression Anxiety Stress Scale 21
FES-1: Falls Efficacy Scale-1
PASE: Physical Activity Scale for the Elderly
IADL: Instrumental Activities of Daily Living
SBP: Systolic blood pressure
DBP: Diastolic blood pressure
HR: Heart rate
SDNN: Standard deviation of NN interval
CV: Coefficient variation
ARV: Average real variability
RMSRV: Root mean square of real variability
LF-nu: Low-frequency normalised unit
HF-nu: High-frequency normalised unit
TP: Total power
LF/HF: Ratio of LF and HF
